# Validation of machine learning models for estimation of left ventricular ejection fraction on point-of-care ultrasound: insights on features that impact performance

**DOI:** 10.1186/s44156-024-00043-2

**Published:** 2024-03-28

**Authors:** Christina L. Luong, Mohammad H. Jafari, Delaram Behnami, Yaksh R. Shah, Lynn Straatman, Nathan Van Woudenberg, Leah Christoff, Nancy Gwadry, Nathaniel M. Hawkins, Eric C. Sayre, Darwin Yeung, Michael Tsang, Ken Gin, John Jue, Parvathy Nair, Purang Abolmaesumi, Teresa Tsang

**Affiliations:** 1 Division of Cardiology, Diamond Health Care Centre 9th Floor Cardiology, University of British Columbia, 2775 Laurel Street, Vancouver, BC V5Z 1M9 Canada; 2 Department of Electrical and Computer Engineering, University of British Columbia, 5500-2332 Main Mall, Vancouver, BC V6T 1Z4 Canada; 3 Faculty of Pharmaceutical Sciences, University of British Columbia, 2775 Laurel Street, Vancouver, BC V5Z 1M9 Canada; 4 British Columbia Centre On Substance Use, 1045 Howe St Suite 400, Vancouver, BC V6Z 2A9 Canada

**Keywords:** Machine learning, Artificial intelligence, Point-of-care ultrasound, Echocardiography, Heart failure

## Abstract

**Background:**

Machine learning (ML) algorithms can accurately estimate left ventricular ejection fraction (LVEF) from echocardiography, but their performance on cardiac point-of-care ultrasound (POCUS) is not well understood.

**Objectives:**

We evaluate the performance of an ML model for estimation of LVEF on cardiac POCUS compared with Level III echocardiographers’ interpretation and formal echo reported LVEF.

**Methods:**

Clinicians at a tertiary care heart failure clinic prospectively scanned 138 participants using hand-carried devices. Video data were analyzed offline by an ML model for LVEF. We compared the ML model's performance with Level III echocardiographers' interpretation and echo reported LVEF.

**Results:**

There were 138 participants scanned, yielding 1257 videos. The ML model generated LVEF predictions on 341 videos. We observed a good intraclass correlation (ICC) between the ML model's predictions and the reference standards (ICC = 0.77–0.84). When comparing LVEF estimates for randomized single POCUS videos, the ICC between the ML model and Level III echocardiographers' estimates was 0.772, and it was 0.778 for videos where quantitative LVEF was feasible. When the Level III echocardiographer reviewed all POCUS videos for a participant, the ICC improved to 0.794 and 0.843 when only accounting for studies that could be segmented. The ML model's LVEF estimates also correlated well with LVEF derived from formal echocardiogram reports (ICC = 0.798).

**Conclusion:**

Our results suggest that clinician-driven cardiac POCUS produces ML model LVEF estimates that correlate well with expert interpretation and echo reported LVEF.

## Introduction

Heart failure (HF) is a serious and increasingly prevalent condition associated with significant morbidity and mortality worldwide. The diagnosis and management of heart failure require the reliable and recurrent evaluation of left ventricular ejection fraction (LVEF) as a representation of LV systolic function, commonly assessed with cardiac ultrasound, also known as echo. An echo is usually performed in the laboratory setting on large full-functionality machines by professional sonographers and often involves the acquisition of up to 150 videos to comprehensively analyze cardiac structure and function. Given the resource-intensive nature of echo, inappropriately prolonged investigation wait times are becoming more common. Mechanisms to improve access to cardiac ultrasound are needed to support the existing healthcare infrastructure.

Machine learning algorithms (ML) have been shown to estimate the LVEF from echocardiography with a high degree of accuracy [1–7]. However, there are few studies that validate the performance of ML models for the prediction of LVEF on cardiac POCUS [8, 9]. Cardiac POCUS imposes challenges additional to those of cart-based echocardiography, further complicating the quantification of cardiac indices such as LVEF. First, cardiac POCUS studies tend to produce images of often inferior quality compared to cart-based echocardiography due to the limited image enhancement capabilities of portable devices [10], patient instability [11], and variable scanner experience.

Our group has previously presented machine learning models for the automated estimation of LVEF in several works [2–4, 12, 13]. We have shown accuracy through testing on POCUS videos scanned by trained sonographers [14]. Validation of ML models on clinician driven POCUS can enable broader use of POCUS, improve access to cardiac ultrasound for LVEF evaluation and may reduce demand for echocardiography.

In this paper, we test our ML LVEF model on heart failure patients' cardiac POCUS videos acquired by clinicians with varying scanning experience. We aim to demonstrate the feasibility and reliability of ML-augmented LVEF estimation on POCUS and compare its performance to blinded level III echocardiographer interpretation and reported LVEF on echocardiogram.

## Material and methods

### Study design and setting

The study procedure and protocols were designed in accordance with the Declaration of Helsinki and received approval from the University of British Columbia institutional review board. Written informed consent for study participation was obtained from all subjects.

#### Subjects

The study recruited participants from the Heart Failure (HF) clinic at a large, academic referral hospital and included individuals with both reduced and preserved ejection fraction.

#### Imagers

The study included 7 physicians and 2 nurse practitioners as clinician scanners, who received training in the operation of the hand-carried ultrasound device (Clarius Scanner PA HD, Clarius Mobile Health Corp; Burnaby, Canada). The physicians were heart failure specialists meeting the criteria of level II echocardiographers, though they did not interpret echocardiography as a part of their usual duties. A level II echocardiographer has acquired 24 weeks of dedicated training and is deemed independent in echo image acquisition and interpretation. The nurse practitioners had no baseline ultrasound experience and were individually trained for the acquisition of the parasternal long axis (PLAX), apical 2 chamber (AP2), and apical 4 chamber (AP4) views on their first 10 participants. All clinician scanners had access to hardcopy and electronic resources for optimal acquisition of the target cardiac views.

#### Data acquisition protocol

Participants were recruited between February 2021 and June 2022 at the HF clinic, which specializes in the evaluation and treatment of heart failure patients. Eligible participants were 18 years of age or older and had undergone an echocardiogram within 3 months of the visit date or had a scheduled echocardiogram. Clinician scanners independently obtained the target views with a level III echocardiographer present to identify unexpected critical findings. The acquired clips were transferred to a regional imaging database for offline analysis by the ML model for view classification and LVEF estimation.

#### ML result analysis protocol

**Fig. 1:**
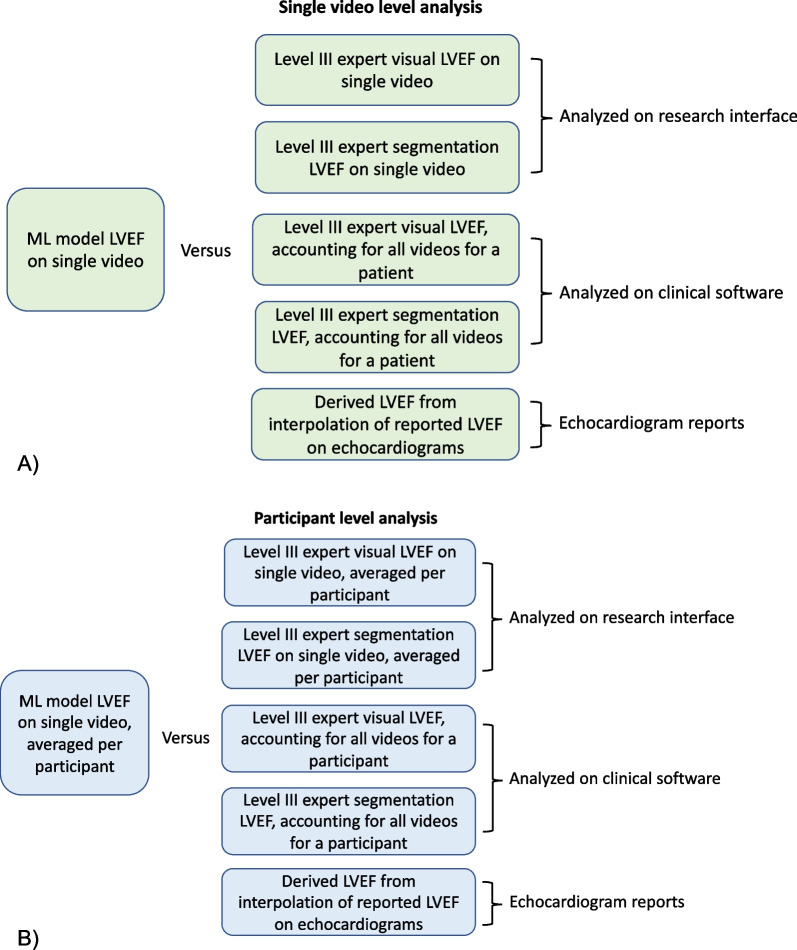
Summary of comparisons and raters. **A** The intraclass correlation coefficient (ICC) was calculated for the ML model estimated left ventricular ejection fraction (LVEF) of single videos as compared with the aforementioned five reference standards. The reference standards were established by a level III echocardiographer by 4 different methods and the derived LVEF using formal echocardiogram reports. **B** The ML model LVEF was calculated at the participant level by averaging the single video estimates for LVEF. The participant ML model LVEF was used for ICC calculation by comparing with 5 reference standards; 2 methods which required averaging of expert LVEF estimates across single videos

For this study, we established five reference standards for comparison which accounted for both the single video and participant level data (Fig. 1).

The level III echocardiographer evaluated LVEF at a single video and participant level; blinded to the clinical data and ML model estimate of LVEF. A level III echocardiographer describes a cardiologist who has obtained the highest level of expertise in echocardiography through a dedicated fellowship entailing 76 weeks of cumulative training. The original study design called for same-day echocardiogram and HF clinic POCUS to allow for direct comparison of LVEF data. However, due to the COVID-19 pandemic, there was decoupling of the clinic visit and echocardiogram, resulting in delays in echocardiogram performance. To account for the difference in timing of the research POCUS and formal echocardiogram, we calculated derived LVEF based on the linear interpolation of values from the echo report before and after the POCUS.

### Machine learning model architecture

The ML model used in this study was previously developed and validated using 2,920 apical echo cines from 2,127 patients. The model is based on U-Net architecture predicting LV segmentation mask and two landmark heatmaps, namely LV apex and mitral valve. The LV mask and the corresponding landmark points are used to estimate LV volume following method of disk on AP4 and AP2 chamber clips. The model is applied to the echo cine frame by frame to obtain the predictions for the entire cardiac cycle. Model architecture and performance were previously described by Jafari *et al* [14].

### Study outcomes and data collection

Clinicians obtained AP2, AP4, and PLAX views during POCUS, aiming to do so within 5 min. Other views could be obtained as needed. A level III echocardiographer was present to alert the clinician to time-sensitive findings. Structured data including age, height, weight, sex, heart rate, blood pressure, clinician scanner type, and cardiomyopathy type were collected. Participant rhythm was obtained from ECG reports. The primary outcome was the intraclass correlation coefficient (ICC) between the ML model LVEF and reference standards at single video and participant levels. Subgroup analysis was performed based on clinical factors including BMI, rhythm, and scanner type.

### Data processing and labelling

The anonymized video data was securely transferred to a research imaging repository after cardiac POCUS completion. A level III echocardiographer (CL) analyzed the blinded videos in two ways (Fig. 1). The first approach estimated LVEF per video file on a research platform, while the second approach produced an overall participant LVEF after viewing all videos using a the clinical application syngo Dynamics (version 20). The echocardiographer would give an LVEF estimate using AP4 and AP2 images visually and Simpson method of disks when feasible. The videos were processed for ML model analysis by cropping with an in-house algorithm, downsizing to 128 × 128 pixels with 30 sampled frames, and rescaling pixel intensities. LVEF estimation required images that were of sufficient quality to enable LV segmentation by the ML model for 30 consecutive frames; videos that did not meet this criteria were excluded.

### Statistical analysis

LVEF estimation was analyzed using ICCs for pairs of individual video LVEF estimates and average LVEF estimates per participant, as described in Fig. 1. Subgroup analysis was performed based on sex, body mass index, rhythm, and scanner type. The analyses were conducted using SAS v9.4 (SAS Institute, Cary, North Carolina). ICC values below 0.5, between 0.5 and 0.75, between 0.75 and 0.90, and above 0.90 represent poor, moderate, good, and excellent reliability, respectively [15].

## Results

### Acquired data sets

There was a total of 138 participants scanned for this study which yielded 1257 videos for analysis. Participant characteristics are summarized in Table 1. Tables 2, 3 provides a synopsis of the POCUS video data for each rating method.

**Table 1:** Participant demographic data

Characteristics	Proportion
Male	118*/138 (85.5%)
Scanned by nurse	91/138 (65.9%)
Scanned by physician	47/138 (34.1%)
Rhythm atrial fibrillation or atrial flutter at the time of scan	54/138 (39.1%)
LVEF > 50%	27/138 (19.6%)
Type of cardiomyopathy • NICMO • ICMO • Unknown	Type of cardiomyopathy • 73/138 (52.9%) • 53/138 (38.4%) • 12/138 (8.7%)

Variable	Mean ± SD
Age (y)	66.2 ± 14.3
Weight (kg)	81.4 ± 18.6
BMI (kg/m^2^)	27.0 ± 5.5
Heart rate at time of scan (BPM)	73.9 ± 16.6
Systolic BP (mmHg)	121.7 ± 19.8
Diastolic BP (mmHg)	68.7 ± 10.1

^*^1 individual identified as a transgender man, analyzed as female sex for this study

**Table 2:** Single video imaging data split by type of rater

Rater	Number of videos assigned an LVEF	Number of videos of insufficient quality for LVEF estimation	Mean estimation of LVEF ± SD
ML model	341	916	0.39 ± 0.13
Level III expert visual LVEF on randomized single videos	851	406	0.41 ± 0.13
Level III expert segmentation LVEF on randomized single videos	245	1012	0.40 ± 0.14
Level III expert visual LVEF, accounting for all videos for a participant	1175*	82	0.40 ± 0.13
Level III expert segmentation LVEF, accounting for all videos for a participant	754^#^	503	0.41 ± 0.13
Derived LVEF from echo reports	N/A	N/A	0.39 ± 0.12

^*^All videos for a participant were included in this category if at least one video in the study was assigned an LVEF by visual assessment

^#^All videos for a participant were included in this category if at least one video in the study was assigned an LVEF by segmentation

**Table 3:** Participant imaging data split by type of rater

Rater	Number of studies assigned an LVEF	Number of studies of insufficient quality for LVEF estimation	Mean estimation of LVEF ± SD
ML model	91	47	0.39 ± 0.11
Level III expert visual LVEF on randomized single videos, averaged per patient	120	18	0.40 ± 0.13
Level III expert segmentation LVEF on randomized single videos, averaged per patient	67	71	0.40 ± 0.14
Level III expert visual LVEF, accounting for all videos for a participant	124	14	0.40 ± 0.13
Level III expert segmentation LVEF, accounting for all videos for a participant	72	66	0.40 ± 0.12
Derived LVEF from echo reports	138	0	0.39 ± 0.12

### Safety

For one participant, the echocardiographer was required to facilitate in clarification of an LV structure that could represent a mass, thrombus, or prominent papillary muscle. POCUS performed by the echocardiographer could not exclude a mass or thrombus therefore, the echocardiographer expedited a formal echocardiogram which confirmed that the structure was a prominent papillary muscle.

### Relationship between ML model and reference rater LVEF estimation

**Fig. 2:**
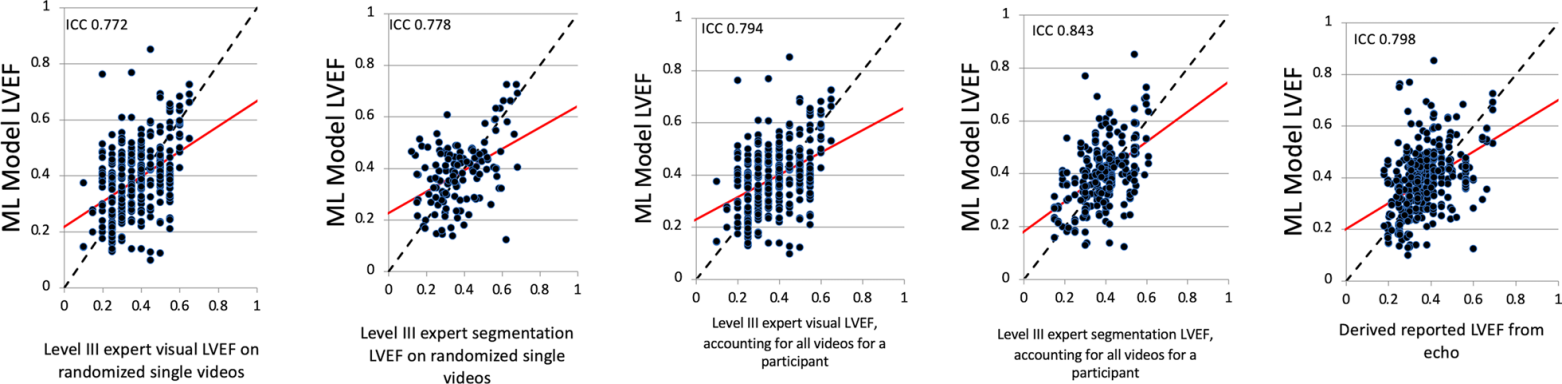
Linear regression plots comparing the ML model to the reference standards. The intraclass correlation coefficient (ICC) for ML model LVEF and level III echocardiographer LVEF was 0.772 [0.501,1.000] and 0.778 [0.578,1.000] for randomized single videos by visual estimate and segmentation, respectively. The ICC for single video ML model LVEF and level III echocardiographer LVEF was 0.794 [0.173, 1.000] for visual assessment and 0.843 [0.310, 1.000] by segmentation when the expert was able to review all clips for a participant. The ICC for ML model LVEF and derived reported LVEF was 0.798 [0.143, 1.000]

Out of a total of 1257 cardiac POCUS videos, 341 were of sufficient quality for ML model estimation of LVEF whereas the level III echocardiographer was able to assign an LVEF to 851 videos by visual assessment and 245 by segmentation. On a randomized single video level, the ICC for ML model and level III echocardiographer LVEF was 0.772 [0.501,1.000] for visual estimates and 0.778 [0.578,1.000] when segmentation was feasible (Table 4). If comparing single videos (accounting for all videos for a participant), the ML model and level III visual LVEF ICC was 0.794 [0.173, 1.000] and improved to 0.843 [0.310, 1.000] when segmentation was possible (Table 4). The ML model LVEF also agrees with the derived LVEF from interpolation of reported LVEF on formal echocardiograms at 0.798 [0.143, 1.000]. The ICC values presented above indicate a good level of inter-rater agreement between the ML model and several reference standards. Figure 2 provides a graphical representation of these relationships.

**Table 4:** Inter-rater agreement for single video data

Observation	Rater 1 of LVEF	Rater 2 of LVEF	ICC (95% CI)
1	ML model	Level III expert visual LVEF on randomized single videos	0.772 (0.501, 1.000)
2	ML model	Level III expert segmentation LVEF on randomized single videos	0.778 (0.578, 1.000)
3	ML model	Level III expert visual LVEF, accounting for all videos for a participant	0.794 (0.173, 1.000)
4	ML model	Level III expert segmentation LVEF, accounting for all videos for a participant	0.843 (0.310, 1.000)
5	ML model	Derived LVEF from echo reports	0.798 (0.143, 1.000)

To analyze the ML model performance at a participant level, LVEF estimates were averaged over all videos acquired for a particular person. The ICC was only 0.344 when the reference standard was mean level III echocardiographer LVEF on randomized single videos and 0.273 on mean LVEF by segmentation. When accounting for all videos for a participant, level III visual LVEF and mean ML model LVEF had an ICC of 0.386 and 0.574 if segmentation was possible. The ICC for mean ML model LVEF and derived LVEF from interpolation of reported LVEF on formal echocardiograms was 0.482.

### Secondary results

The impact of body mass index (BMI), atrial fibrillation (AF)/atrial flutter (AFL), sex, and scanner type (physician or nurse) on ML model performance was also examined. On the individual video level, the correlations between the ML model and reference standards was 0.813–0.909 for BMI ≥ 30 and 0.709–0.802 for BMI < 30. Comparing the derived echocardiogram LVEF estimation to the ML model saw an ICC of 0.870 for BMI ≥ 30 and 0.741 for BMI < 30 (Table 5). The ICC between these BMI groups is relatively similar and represents good inter-rater reliability between the ML model and reference standards.

**Table 5:** Inter-rater agreement for single video data, subgroup analyses

Effect of BMI on LVEF estimation. BMI ≥ 30 or BMI < 30				
Observation	Rater 1 of LVEF	Rater 2 of LVEF	ICC (95% CI) BMI ≥ 30 (n = 80)	ICC (95% CI) BMI < 30 (n = 260)
1	ML model	Level III expert visual LVEF on randomized single videos	0.813 (0.247, 1.000)	0.749 (0.740, 1.000)
2	ML model	Level III expert segmentation LVEF on randomized single videos	0.829 (0.165, 1.000)	0.709 (0.098, 1.000)
3	ML model	Level III expert visual LVEF, accounting for all videos for a participant	0.822 (0.129, 0.999)	0.771 (0.551, 1.000)
4	ML model	Level III expert segmentation LVEF, accounting for all videos for a participant	0.909 (0.481, 1.000)	0.802 (0.243, 1.000)
5	ML model	Derived LVEF from echo reports	0.870 (0.610, 1.000)	0.741 (0.071, 1.000)

Effect of sex on LVEF estimation: male or female				
Observation	Rater 1 of LVEF	Rater 2 of LVEF	ICC (95% CI) male (n = 293)	ICC (95% CI) female (n = 49)
1	ML model	Level III expert visual LVEF on randomized single videos	0.693 (0.089, 1.000)	0.901 (0.520, 1.000)
2	ML model	Level III expert segmentation LVEF on randomized single videos	0.705 (0.073, 1.000)	0.869 (0.293, 1.000)
3	ML model	Level III expert visual LVEF, accounting for all videos for a participant	0.740 (0.067, 0.999)	0.877 (0.503, 1.000)
4	ML model	Level III expert segmentation LVEF, accounting for all videos for a participant	0.796 (0.176, 1.000)	0.901 (0.477, 1.000)
5	ML model	Derived LVEF from echo reports	0.758 (0.131, 1.000)	0.859 (0.279, 1.000)

Effect of atrial fibrillation (AF) or atrial flutter (AFL) on LVEF estimation				
Observation	Rater 1 of LVEF	Rater 2 of LVEF	ICC (95% CI) AF or AFL (n = 108)	ICC (95% CI) Non-AF or non-AFL (n = 234)
1	ML Model	Level III expert visual LVEF on randomized single videos	0.684 (-0.143, 1.000)	0.809 (0.346, 1.000)
2	ML Model	Level III expert segmentation LVEF on randomized single videos	0.596 (-0.067, 0.999)	0.829 (0.135, 0.999)
3	ML Model	Level III expert visual LVEF, accounting for all videos for a participant	0.708 (0.182, 1.000)	0.831 (0.210, 1.000)
4	ML model	Level III expert segmentation LVEF, accounting for all videos for a participant	0.823 (0.350, 1.000)	0.860 (0.428, 1.000)
5	ML Model	Derived LVEF from echo reports	0.673 (-0.043, 1.000)	0.841 (0.271, 1.000)

Effect of scanner on LVEF estimation: physician (MD) vs Nurse Practitioner (N)				
Observation	Rater 1 of LVEF	Rater 2 of LVEF	ICC (95% CI) MD (n = 95)	ICC (95% CI) N (247)
1	ML model	Level III expert visual LVEF on randomized single videos	0.582 (−0.015, 0.997)	0.800 (0.154, 1.000)
2	ML model	Level III expert segmentation LVEF on randomized single videos	0.649 (−0.028, 0.999)	0.810 (0.373, 1.000)
3	ML model	Level III expert visual LVEF, accounting for all videos for a participant	0.550 (−0.020, 0.997)	0.845 (0.784, 1.000)
4	ML model	Level III expert segmentation LVEF, accounting for all videos for a participant	0.569 (−0.015, 0.995)	0.884 (0.490, 1.000)
5	ML model	Derived LVEF from echo reports	0.657 (−0.003, 0.998)	0.840 (0.715, 1.000)

We examined the impact of sex on ML model LVEF estimation and found ICC values of 0.693–0.796 and 0.869–0.901 for males and females, respectively (Table 5). The ML model and derived echocardiogram LVEF had ICC values of 0.758 and 0.859 for males and females, respectively. The reference standards and ML model results show a good/excellent inter-rater reliability for females and moderate/good inter-rater reliability for males. There was 1 transgendered man whose data would have been analyzed as female, however; the images acquired were of insufficient quality for ML model analysis.

We also performed analysis for AF/AFL and non-AF/AFL rhythms (sinus, paced, other). On the single video level, the correlations between the ML model reference standards was 0.596–0.823 for AF/AFL and 0.809–0.860 for non-AF/AFL rhythms (Table 5). Unlike the BMI result, there seems to be a greater degree of variability in the ICC based on rhythm and reference rater type. The best ML model performance by ICC was with level III segmentation LVEF when accounting for all videos for a participant; this yielded an ICC of 0.823 for those in AF/AFL and 0.860 for those in non-AF/AFL rhythms. The worst ML model performance by ICC was with level III segmentation LVEF on randomized single videos; this yielded an ICC of only 0.596 (moderate correlation) for those in AF/AFL and 0.829 (good correlation) for those in non-AF/AFL rhythms. The ICC for derived echocardiogram LVEF estimation and ML model demonstrated an ICC of 0.673 for AF/AFL and 0.841 for non-AF/AFL. These results suggest that the AF/AFL rhythm reduces the accuracy of ML model predictions of LVEF compared to expert assessment.

**Fig. 3:**
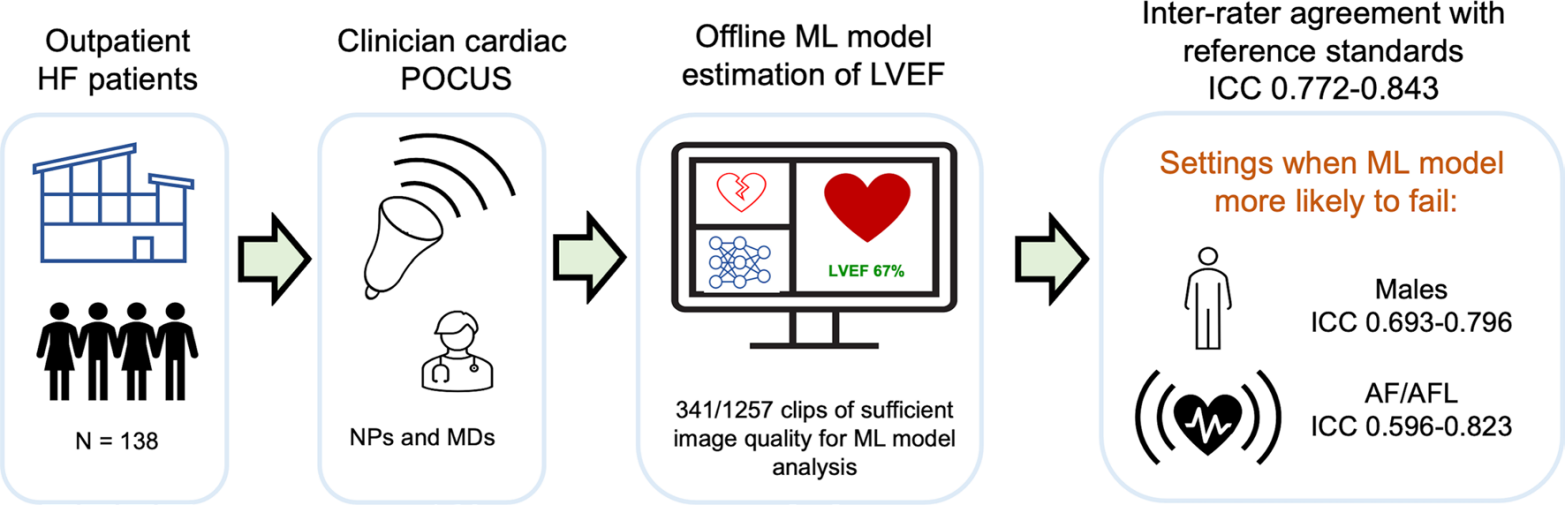
Central illustration: performance of machine learning model for left ventricular ejection fraction on clinician scanned point of care ultrasound in heart failure clinic. *AF/AFL* atrial fibrillation/flutter, *HF* heart failure, *ICC* intraclass correlation, *LVEF* left ventricular ejection fraction, *ML* machine learning, *POCUS* point of care ultrasound

Lastly, we compared the ML model performance based on scanner type; physician (level II echocardiographer) versus nurse (novice scanner). Unexpectedly, the ICC value for ML model LVEF for physician scans was only 0.550–0.649 (moderate correlation) compared with 0.800–0.884 (good correlation) for nurse scanned videos (Table 5). The ML model and derived echocardiogram had ICC values of 0.657 and 0.840 for physician and nurse scans, respectively. These results indicate that when the POCUS was conducted by the physician compared to nurses, there was higher disagreement between the ML model's and reference rater’s estimated LVEF. The overall study results are outline in the Fig. 3.

## Discussion

Our study evaluated the performance of an ML model for the prediction of LVEF on cardiac POCUS videos obtained by clinicians and demonstrated: (i) the ML model conveys a good degree of correlation with expert-estimated LVEF and echocardiogram reported LVEF and (ii) clinical factors may influence model performance. Although we demonstrated good correlation when images were of adequate quality, we acknowledge that most images were not of sufficient quality for analysis (ML model LV segmentation for 30 consecutive frames). This is likely a reflection of the data composition, with the majority of scans performed by less experienced scanners (nurse practitioners) who were particularly keen to enroll participants in this study.

Although previous studies have examined the accuracy of other ML models for LVEF estimation, they did not utilize clinician driven POCUS and instead focused on echocardiogram data [1, 16, 17] or imaging performed by sonographers [14]. Asch et al., showed good agreement in an ML model estimation of LVEF compared to reference values on cardiac POCUS. However the majority of clips were acquired by sonographers (protocol 1), with only a subset scanned by nurses facilitated by an artificial intelligence powered scanning software, not widely available for routine use (protocol 2) [9]. Furthermore, this study did not examine clinical features that may influence model performance to give insight on considerations for use. The impact of imaging differences in clinical POCUS as compared to echocardiography should not be underestimated. Crockett et al. applied a “best-in-class” echocardiography trained ML model, EchoNet-Dynamic, to a retrospective collection of cardiac POCUS studies and found suboptimal ML model performance with an AUC of only 0.74 versus the published benchmark of 0.97 for the classification of LVEF < 50% [8]. Our model fared slightly better with ICC of 0.77 to 0.84 but was similarly impacted by issues regarding image quality related to scanner and clinical factors.

Our study had robust clinical characterization of the cohort and we utilized 5 forms of reference standards to capture the uncertainty associated with LVEF analysis on cardiac ultrasound. Part of the rationale for multiple reference standards was due to the reduction in access to same day formal echocardiography during the COVID-19 pandemic. In lieu of same day formal echocardiogram, we had a level III echocardiographer’s blinded visual and segmentation-based estimation of the LVEF on POCUS videos as the reference standards. The range in the ML model’s ICC with the various reference standards indicates the clinical challenge of consistent LVEF estimation based on single videos. The design of our study allowed for evaluation of the ML model’s performance on a cohort with a high prevalence of arrhythmia, elevated BMI, and imaging by novice scanners, which may better reflect real-world settings. Furthermore, the well characterized cohort enabled subgroup analyses to delineate conditions that may contribute to model failure.

When examining ML model performance by type of reference rater and subgroup, some interesting observations emerge. Although the ML model performed well relative to expert annotation for individual video files, the same cannot be said when the model was applied to the participant level. When the LVEF was averaged across all the videos for an individual, the ICC notably deteriorated. This suggests that there is a substantial difference in the appearance of LVEF between videos, likely a reflection of poor and/or inconsistent image quality. This was seen most prominently in the ML model comparisons with randomized single videos when the expert was forced to assign an LVEF without the context of other clips. The ICC is slightly better if the reference standard is level III LVEF accounting for all videos for a participant as this allows the expert to assign an LVEF that applies to all images based on the summation of data. This is similar to clinical practice where the expert is likely assigning an overall study LVEF, applying a heavier weighting to videos deemed most valid and discounting videos of poorer quality.

Co-morbid conditions such as obesity and atrial fibrillation and factors like sex of the patient and the qualifications of the scanner have been known to affect the quality of the point of care ultrasound image [18, 19]. As poorer image quality can reduce the accuracy of the LVEF estimation for all types of raters, we investigated the effects of BMI, sex, atrial fibrillation or atrial flutter, and the type of scanner on interpretation of the LVEF. Siadecki et al., showed that the quality of cardiac POCUS images decreases as BMI increases [20] though this did not seem to be associated with lower ICC in our study. On the other hand, videos while in AF or AFL correlated with lower ICC values. Although there is very limited data regarding the impact of AF/AFL on validity and reproducibility of LVEF estimation on echocardiogram [21], our study demonstrate that the rhythm likely plays a role in accuracy of ML model estimation of LVEF which may also impact human interpretation.

Notably, when the POCUS was scanned by nurses, the ML model had higher agreement to the reference standard than when the POCUS was scanned by a physician. This unexpected finding was examined more closely with characterization of the cohort for which the clips were obtained. There were 342 video files that were of adequate quality for ML LVEF estimation. Of these, approximately 28% were obtained by physicians. Closer examination of the cohort demonstrated significant differences that may explain this finding. The proportion of videos of patients with BMI < 30 were similar between nurse and physician scans (76–77%); however, physician videos were more likely to be obtained from patients with AF/AFL as compared to the nurse scanned clips (41% vs. 28%, p = 0.01). Heart rate and diastolic blood pressure were also significantly higher in the physician cohort which may reflect a less stable patient population. Patients who are less optimized from a heart failure point of view may have difficulty participating in maneuvers such as breath-holding or laying in the left lateral decubitus position for optimal scanning. The irregularity of heart rate with atrial fibrillation also decreases the accuracy of expert and ML model LVEF estimation.

As demand for echocardiography increases with an aging population, the need for accessible cardiac imaging has become more pronounced. The ML model examined in this study is computationally light weight and can be loaded onto portable and hand carried ultrasound platforms for rapid and automated estimation of LVEF. ML augmented interpretation of cardiac ultrasound is an opportunity to evaluate LV function in between formal imaging, reduce the demand for formal echo, and/or facilitate triaging of echo request. ML estimation of LVEF on POCUS devices can guide treatment decisions at the bedside, potentially expediting care, reducing costs within the health care system, and improving the patient experience.

This study has several limitations. Although our cohort represents one of the largest cardiac POCUS studies for validation of an ML model the sample size is relatively modest. We allowed for clinician scanners with a range of experience to reflect real-world imaging, however, this did result in many videos that were insufficient for analysis. The low number of videos feasible for ML estimation may reduce the generalizability of our study results. The low-quality imaging obtained on cardiac POCUS, due to variable expertise and patient factors, will likely remain a major pain point for the widespread use of cardiac POCUS and by extension ML augmented POCUS.

Future studies with more participants and higher quality videos can further support the use of the ML model in clinical settings. Furthermore, the ML model does not test other parameters of LV function besides LVEF such as stroke volume and cardiac output. External validation will also be essential for the clinical application of our model. As with all ML models vying for application in clinical settings, the outputs should not be used as the sole factor that directs therapy. ML outputs should be regarded as a component among many that should be taken in consideration and integrated by a skilled clinician.

## Conclusion

In summary, we demonstrate that our ML model is able to estimate LVEF on cardiac POCUS images from a cohort of heart failure patients with good inter-rater reliability (ICC = 0.77 to 0.84) compared with several reference standards. However, image quality and clinical factors including atrial fibrillation/flutter have adverse impact on ML model analysis feasibility and performance and should be considered when applying ML models for clinical use.

### Clinical perspectives

Machine learning models that have been trained and tested on echocardiogram data for predicting LVEF (Left Ventricular Ejection Fraction) can be successfully applied to clinician-driven point-of-care ultrasound. However, the quality of the ultrasound images is a major limitation for broad application, and in certain settings, such as arrhythmia, the model may fail.

## Data Availability

The data that support the findings of this study are available on request from the corresponding author, CL. The data request is subject to approval by the University of British Columbia Clinical Research Ethics Board.

## Abbreviations

AF/AFL: Atrial fibrillation/atrial flutter
BMI: Body mass index
AP2: Apical 2 chamber view
AP4: Apical 4 chamber view
HF: Heart failure
ICC: Intraclass correlation
LVEF: Left ventricular ejection fraction
ML: Machine learning
PLAX: Parasternal long-axis view
POCUS: Point of care ultrasound
